# Illustrated Skin Diseases, an Atlas and Text Book, with Special Reference to Modern Diagnosis and the Approved Methods of Treatment

**Published:** 1898-10

**Authors:** 


ILLUSTRATED SKIN DISEASES, an Atlas and Text Book, with Special Reference to Modern Diagnosis and the Approved Methods of Treatment. By WILLIAM S. GOTTHEIL, M. D., Professor of Skin and Venereal Diseases at the New York School of Clinical Medicine; formerly Lecturer on Dermatology in the New York Polyclinic; Consulting Dermatologist to the Orphan Asylum of the Sheltering Guardian Society; Dermatologist to the Lebanon Hospital, the Northwestern and the West Side German Dispensaries; Fellow of the New York Academy of Medicine and Member of the New York County Medical Society, etc. New York: E. B. Treat & Co., Publishers.

Portfolios I., II., III. of this important work are in our hands, the first of which deals with the anatomy and physiology of the skin. This section is well arranged, quite exhaustive and comprehensive. The illustrations exhibit special merit, having been selected and depicted with the greatest care.

Portfolio II. is devoted to therapeutics of the skin, classification, and describes the functional disorders: Pruritus, hyperidrosis, chromidrosis and bromidrosis. That part referring to therapeutics is handled with skill and accuracy, being full of detail and eminently practical. The classification adopted is that of Jessner, somewhat modified; the choice was made, avowedly, for reasons of convenience, for at the present day, like that of Hebra, it cannot be defended on any other ground.

In addition to the maladies described in section No. 2, Portfolio III. contains a description of seborrhea, comedo, milium, sebaceous cyst, asteatosis, erythema simplex, livedo, urticaria, prurigo and purpura. In each case a concise exposé of our present state of knowledge regarding them is accurately given, being especially complete in diagnosis and treatment.

The colored photographs employed, necessary to the elucidation of the text, are of unusual interest and must be studied to be appreciated. We cordially commend the work to our readers. E. W.

